# Regulated Expression of PTPRJ by COX-2/PGE_2_ Axis in Endothelial Cells

**DOI:** 10.1371/journal.pone.0114996

**Published:** 2014-12-22

**Authors:** Xiaobing Xu, Wenya Lan, Xinxin Jin, Bin Wang, Hongbo Yan, Xi Chen, Xiaowei Lai, Li Zhang, Xiaohua Zhang, Zhaoshen Li

**Affiliations:** 1 Department of Gastroenterology and Hepatology, Jinling Hospital, Clinical School of Nanjing, Second Military Medical University, Nanjing, China; 2 Department of Gastroenterology, Changhai Hospital, Second Military Medical University, Shanghai, China; 3 Department of Geriatric Neurology, Nanjing Brain Hospital Affiliated to Nanjing Medical University, Nanjing, China; 4 State Key Laboratory of Proteomics, Beijing Proteome Research Center, Beijing Institute of Radiation Medicine, Beijing, China; Chang Gung University, Taiwan

## Abstract

**Background:**

This study was designed to examine a novel role of COX-2/PGE_2_ signaling as a regulator of PTPRJ expression in endothelial cells.

**Methods:**

A bioinformatics analysis of a whole genome array was carried out to search for regulators of PTPRJ expression in endothelial cells. PTPRJ expression was also measured in endothelial cells derived from a balloon injury-induced neointimal hyperplasia model in male New Zealand Rabbits. Changes in PTPRJ expression in HUVEC cells was examined by RT-PCR and western blotting after transfection of COX-2 plasmids or treatment with varying concentrations of a COX-2 inhibitor.

**Results:**

A significant correlation was identified between COX-2 and PTPRJ in GSE39264 (Pearson correlation coefficient  = −0.87; n = 22; P<0.01, two-tailed). PTPRJ expression was reduced during the progression of neointimal hyperplasia after balloon injury, which correlated with an increase in COX-2 expression. In HUVECs, after transfection with 1 µg/ml, 0.5 µg/ml, or 0.25 µg/ml COX-2 plasmids, PTPRJ protein expression was reduced to 0.60- (±0.08), 0.75- (±0.09), and 0.88- (±0.04) fold, respectively, while mRNA expression was reduced to 0.15- (±0.03), 0.26- (±0.05), and 0.47- (±0.09) fold, respectively. After treatment of HUVECs with 10 µmol/L or 20 µmol/L celecoxib, the reduction in PTPRJ expression induced by COX-2 over-expression was not only rescued but in fact increased by 2.05-fold (±0.28) and 3.34-fold (±0.37), respectively, compared with control.

**Conclusions:**

Our results suggest that COX-2/PGE2 signaling may function as a negative regulator of PTPRJ expression in endothelial cells both *in vivo* and *vitro*.

## Introduction

Protein tyrosine phosphatase receptor-type J (PTPRJ), encoded by *DEP1*, is a receptor protein containing eight extracellular FNIII repeats and one cytoplasmic catalytic domain, and is expressed quite ubiquitously among different cell types [1]–[3]. Since it has a reduced expression in some malignant tumors it is considered as a putative tumor suppressor, which is further substantiated by its cell density-mediated regulation, and reversion of the transformed phenotype when PTPRJ function is restored [4]–[9].

Currently available evidence indicates that PTPRJ plays a significant role in the regulation of angiogenesis, a key pre-requisite for tumorigenesis and metastatic progression [10]–[12]. This process occurs through signal transduction pathways involving the expression of several receptors and substrates, including PDGF b-receptor [13], hepatocyte growth factor (HGF) receptor [14], vascular endothelial growth factor (VEGF) receptor-2 [15] and the p85 subunit of PI3-kinase [16]. Cyclooxygenase-2 (COX-2), an inflammation-associated enzyme, is an important mediator for tumor initiation in tissues subjected to chronic inflammation [17]. Additionally, it has been established that COX-2 overexpression is both a signature and a primary determinant of tumor progression and metastasis in different cancers [17]–[19]. COX-2 is also overexpressed in many cancers and has been associated with increased VEGF production and angiogenesis [20]. Furthermore, it has been demonstrated that COX-2/PGE2 axis is involved in cancer progression through inactivation of host antitumor immune cells [21], as well as stimulation of tumor cell migration, invasiveness and tumor-associated angiogenesis [22]–[25].

The aforementioned findings prompted us to query whether the expression of PTPRJ during angiogenesis progression is altered, or if the COX-2/PTPRJ axis has a possible role in the initiation and progression of angiogenesis. In this study, we show that PTPRJ can be down-regulated by COX-2/PGE_2_ signaling during angiogenesis. Inhibition of COX-2/PGE2 signaling may hence be a potent mechanism to enhance PTPRJ expression during this process.

## Materials and Methods

### PubMed GEO database accession and expression analysis

GSE39264 performed expression array analysis on RNA isolated directly from the vascular cells of individual control, pre-lesioned, and atherosclerotic mouse aortas. Array expression analysis was not performed on smooth muscle cells or macrophages. RNA from MAEC of pre-lesioned hyperlipidemia and normal-lipidemic mice was isolated, amplified, and arrayed. Mouse aortas were also treated in either media (DMEM) or media containing either LPS, oxLDL, or oxPAPC for 4 hours prior to RNA isolation and amplification.

### Animals and carotid artery balloon injury model

The study protocol was approved by the ethics review board of the Jinling Hospital (2012GJJ-068), and all procedures were carried out in accordance with the Declaration of Helsinki and relevant policies in China outlined by the governmental committee for animal research. Male New Zealand white rabbits, each weighing 2.5–3 grams, were fed with standard laboratory chow and allowed free access to water in an air-conditioned room with a 12 hours light/dark cycle. Before the experiment, animals were housed under these conditions for 7 days to allow acclimatization. Animals were fed a 0.5% cholesterol diet for one week before and four weeks after balloon injury (BI). Animal body weights were recorded prior to and at the end of the experiment at week 4. Animals were anesthetized with 25 mg/kg intravenously (iv) administered pentobarbital.

The carotid artery was de-endothelialized by an angioplasty balloon catheter (diameter, 2.5 mm; length, 10 mm) as described previously [26]. Briefly, the catheter was advanced three times from the left external carotid artery down to the level of the common carotid arteries. The right common carotid of the same rabbit was left as control. To attain a consistent degree of vessel wall injury across all of the animals, we used the same balloon diameter and resistance during withdrawal for each of the rabbits. All procedures were performed by a single operator.

### Cell culture, characterization and transfection

Human umbilical vein endothelial cells (HUVECs), an immortalized non-tumor cell line derived from normal human umbilical vein, were purchased from American Type Culture Collection (ATCC, catalogue number CRL-1730), and cultured at 37°C and 5% CO_2_ in high-glucose Dulbecco's modified Eagle's medium (DMEM) containing 10% fetal bovine serum (FBS; Invitrogen), 100 U/ml penicillin and 100 µg/ml streptomycin.

In order to characterize their morphology, the cells were cultured with 3 µg/ml puromycin for 72 hr and maintained in DMEM with 1.5 µg/ml puromycin. Cell morphology was then examined when the cells reached confluence.

Immunofluorescence was performed to detect the expression of VE-cadherin and Von Willebrand factor in HUVECs, according to standard protocols. HUVECs were transfected using Fugene HP (Promega) according to the manufacturer's instructions. Briefly, 48 hours after transfection, HUVECs were fixed in 4% (w/v) paraformaldehyde for 20 minutes, washed three times with phosphate buffered saline (PBS) and air-dried at 4°C. Following treatment with 5% bovine serum albumin (BSA) for 30 minutes, the cells were incubated for 12 hours at 4°C with either anti-Von Willebrand factor primary antibody (rabbit anti-human; Abcam, UK) or anti-VE-cadherin primary antibody (rabbit anti-human; Cell Signaling Technology, US), and PE-conjugated goat anti-mouse IgG (Santa Cruz, CA, USA) for 1 hour at 37°C. The specific fluorescence was examined using an LSM700 laser scanning confocal microscope (Carl Zeiss, German). At least three independent experiments were performed, and cultures were examined by microscope (Olympus, Japan).

### Complementary DNA (cDNA)constructs

The PTPRJ (*DEP1*) cDNA construct was a kind gift from Dr. Nicholas K Tonks (Cold Spring Harbor Laboratory, NY, USA) [27]. The plasmids encoding COX-2 was a kind gift from Dr. Marta Casado (Institute of Biomedicine of Valencia, Valencia, Spain) [28].

### Western blotting

Harvested carotid artery intima and HUVEC cells were suspended and sonicated in 1 ml ice-cold RIPA lysis buffer (Beyotime Technology, China). Samples were centrifuged at 14,000 g at 4°C for 15 minutes, and the cytosolic fraction was then collected as part of the supernatant and assayed quantitatively with the BCA method. After boiling for 5 minutes, 20–25 µl of the lysate (corresponding to 50 mg of protein) was loaded onto a 10% SDS PAGE gel and run at 80 V for 15 minutes and 120 V for 1 hour. The proteins in the gel were then transferred to a PVDF membrane (Millipore, US). After blocking, the membranes were probed with anti-COX-2 (1∶2000 dilution; Epitomics, UK) and anti-PTPRJ (1∶500 dilution; Abcam, UK), followed by the addition of horseradish peroxidase (HRP)–conjugated secondary antibody (1∶3000 dilution; Sigma, US) at 37°C for 1 hour. Immunoreactive proteins were visualized using a chemiluminescence kit (Immobilon Western Chemiluminescent HRP Substrate; Millipore). Band intensities were determined using the Tanon 4500 chemiluminescent imaging system (Tanon Science & Technology, China) and Image J software (NIH, USA).

### RNA isolation and real-time quantitative PCR

Total RNA was extracted from tissues and cells using Trizol Reagent (Invitrogen, US). The concentration of extracted RNA was determined using an ultraviolet spectrophotometer. cDNA was synthesized from 2 µg of total RNA using PrimeScript RT reagent (Takara Bio, Inc.). The reactions were performed using the SYBR PrimeScript RT-PCR kit (Takara Bio, Inc.) with an ABI 7500 Real-Time PCR Systems (Applied Biosystems) according to a standard protocol. As an internal control, levels of the housekeeping genes ß-actin (*ACTB*) (for rabbit) or *RPLL22* (for HUVECs) were quantified in parallel with the target genes. Crossing threshold (C_t_) values were calculated with the 7500 Fast Real-Time PCR System software, using the second derivative maximum method. Data were analyzed using the 2^−△△*C*T^ method, and all samples were run in triplicate. Primers used for are listed in **Table 1**.

**Table 1:** Primers used for the real time analysis.

Gene	Primers sequences (5′-3′)		Fragment size (bp)	
PTPRJ (*DEP1*) (Rabbit)	GGCTTCGCAGAGGAATACGA	GGAATGGTAGCCAGGCATGT		198
PTPRJ (*DEP1*) (Human)	CTGGAAGAGCCCTGACGGTGC	TTGCTGGGCCGTGTGTACTGTG		207
*COX2*(Rabbit)	TGCTGTTCCAACCCATGTCA	CTGGTCAGAAATTCCGGCGT		125
*COX2*(Human)	CCCTTGGGTGTCAAAGGTAA	GCCCTCGCTTATGATCTGTC		169
*ACTB*(Rabbit)	GGACCTGACCGACTACCTCA	GGCAGCTCGTAGCTCTTCTC		180
*RPL22*(Human)	TCGCTCACCTCCCTTTCTAA	TCACGGTGATCTTGCTCTTG		250

### Enzyme-linked immunosorbent assay

HUVEC supernatants, stored in aliquots at −70°C, were thawed on ice prior to the measurement of PGE_2_ using specific enzyme-linked immunosorbent assay (ELISA) kits purchased from R&D Systems (Minneapolis, MN, USA) according to the manufacturer's instructions. After normalizing to the total protein content of the supernatant, results were expressed as picograms of PGE_2_ per milligrams of total protein. DMEM with 10% FBS was used as control.

### Statistical analysis

All experiments were performed at least three times, and data were expressed as mean ± S.D. Student's *t*-test (one sample or independent samples) and two-way ANOVA were used to compare sample means and to determine statistical significance. Correlation between two continuous variables was determined by the Pearson's correlation test. SPSS 16.0 software was used for data analysis. A value of P<0.05 was statistically considered significant.

## Results

### The correlation of COX-2 and PTPRJ expression from protein expression array analysis

**Figure 1:**
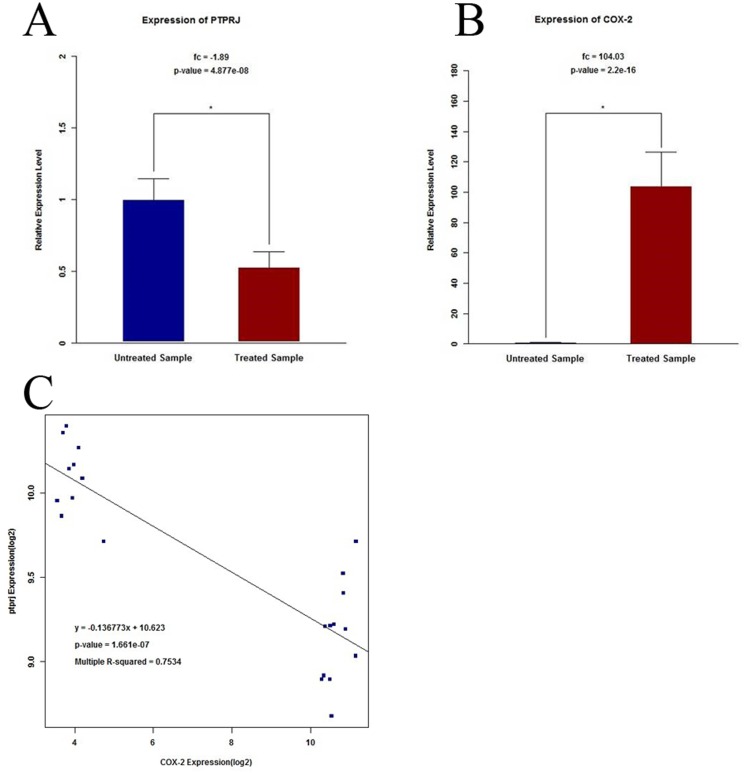
The correlation of COX-2 and PTPRJ expression in aortic endothelial cells from whole-genome array expression analysis GSE39264. (A) Differential expression of PTPRJ from analysis of a MAEC whole-genome array. *P<0.05 versus untreated group. (B) Differential expression of COX-2 from analysis of a MAEC whole-genome array. *P<0.05 versus untreated group. (C) Correlation of COX-2 and PTPRJ expression from analysis of a MAEC whole-genome array. (Pearson correlation coefficient, −0.87; n = 22; P<0.01, two-tailed). Genes were selected from the microarray data using a threshold of 10% FDR (false discovery rate). P values for fold change between untreated and treated MAECs were determined by *t*-test; P values for fold change were adjusted using Benjamini–Hochberg.

Using bioinformatics analysis of the Pubmed GEO database, we identified PTPRJ expression in endothelial cells as GSE39264. Our analysis revealed that COX-2 expression was increased in treated MAECs compared to untreated MAECs, (104.03±0.5, P<0.01 **Fig. 1, A**), while expression of PTPRJ decreased (1.89±0.4, P<0.01 Fig. 1, B). Statistical analysis of these results revealed a significant correlation between COX-2 and PTPRJ expression. (Pearson correlation coefficient, −0.87; n = 22; P<0.01, two-tailed; **Fig. 1, C**).

### Reduced PTPRJ expression in injury-induced neointimal hyperplasia was correlated with increased COX-2 expression

**Figure 2:**
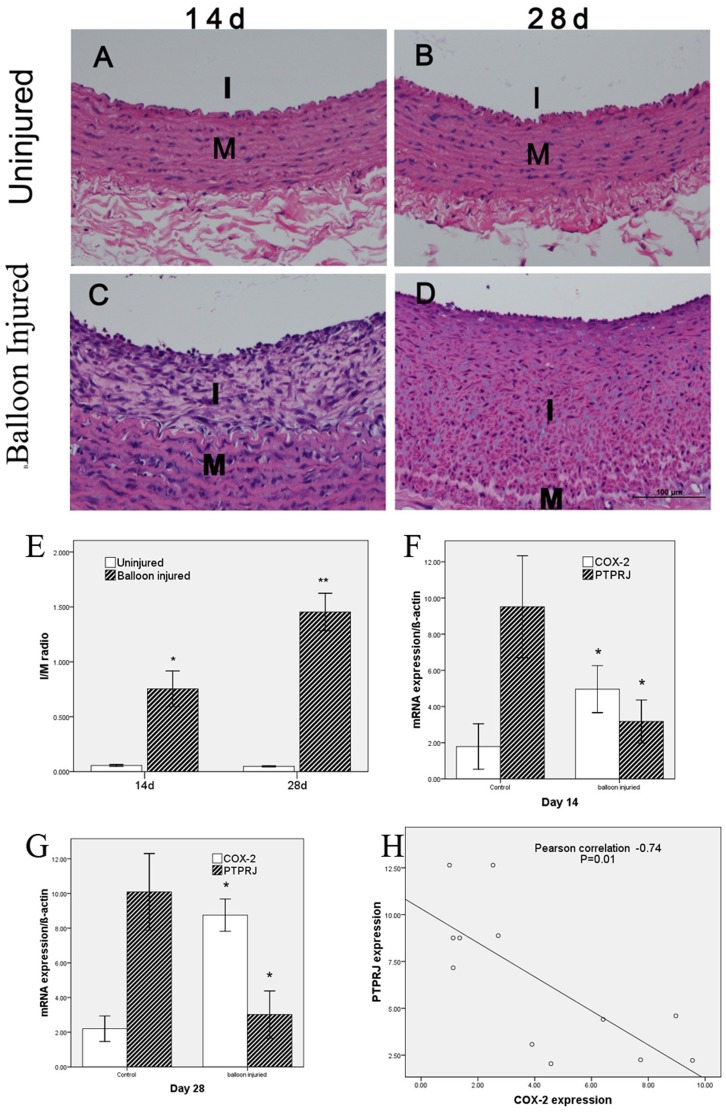
Injury-induced down-expression of PTPRJ is correlated with increased COX-2. (A–D) Representative sections of hematoxylin and eosin-stained arterial samples from control (A and B) and BI (C and D) rabbits. BI significantly induced neointimal hyperplasia. Images acquired at 200X magnification. (E) Morphometric data *P<0.05 versus uninjured group; n = 3 in each group. (F–G) PTPRJ and COX-2 expression quantified by qRT-PCR. Values were normalized to ß-actin expression. *P<0.05 versus uninjured group. (H) Correlation of COX-2 and PTPRJ expression (r = −0.74, P≤0.01).

In order to characterize the role of COX-2/PGE_2_ signaling in the development of neointimal hyperplasia after vascular injury, we developed the rabbit carotid artery BI model. As shown in **Fig. 2, A–D**, at 14 and 28 d following BI, the arteries displayed dramatic neointimal hyperplasia (**Fig. 2, C** and **D**). Additionally, we observed an increased I/M ratio, which represents the extent of hyperplasia in balloon-injured arteries relative to the uninjured arteries of the same animal (14 d: 0.75±0.16 versus 0.06±0.01, P = 0.02; 28 d: 1.45±0.17 versus 0.05±0.01, P = 0.01; **Fig. 2**, E). This result was accompanied by increased expression of COX-2 (BI versus control, 14 d: 4.96±1.30 versus 1.79±1.23; 28d: 8.75±0.93 versus 2.20±0.74 **Fig. 2, F** and **G**), and decreased expression of PTPRJ in the hyperplastic artery intima (BI versus control, 14 d: 3.17±1.19 versus 9.52±2.82; 28 d: 3.02±1.36 versus 10.09±2.21; **Fig. 2, F** and **G**). Moreover, we found a significant correlation between COX-2 and PTPRJ expression (Pearson correlation coefficient, −0.74; n = 12; P<0.01, two-tailed; **Fig. 2, H**).

### Characterization of HUVECs

**Figure 3:**
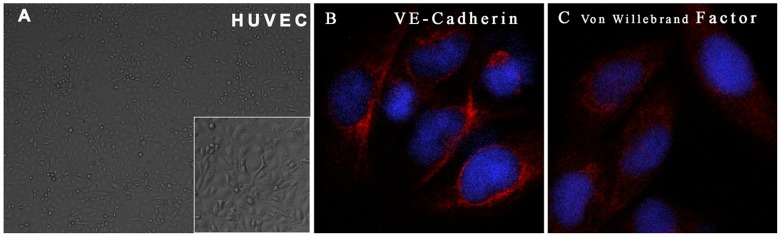
Characterization of HUVECs by morphology and immunofluorescence. (A) Confluent cells exhibited typical cobblestone morphology; insert image at 200X magnification. (B and C) Immunofluorescence of samples labeled for VE-cadherin (B) or Von Willebrand factor (C). The immunoreactive positive cells were stained red and imaged at 400X magnification.

As shown in **Fig. 3 A**, the typical cobblestone morphology of HUVECs was observed in cells reaching confluence, and immunofluorescence staining revealed localization of VE-Cadherin and Von Willebrand factor in the cell membrane and cytoplasm, respectively (**Fig. 3 B** and **C**), suggesting that the cells were endothelial cells.

### Over-expression of COX-2 suppresses PTPRJ expressing in HUVECs

**Figure 4:**
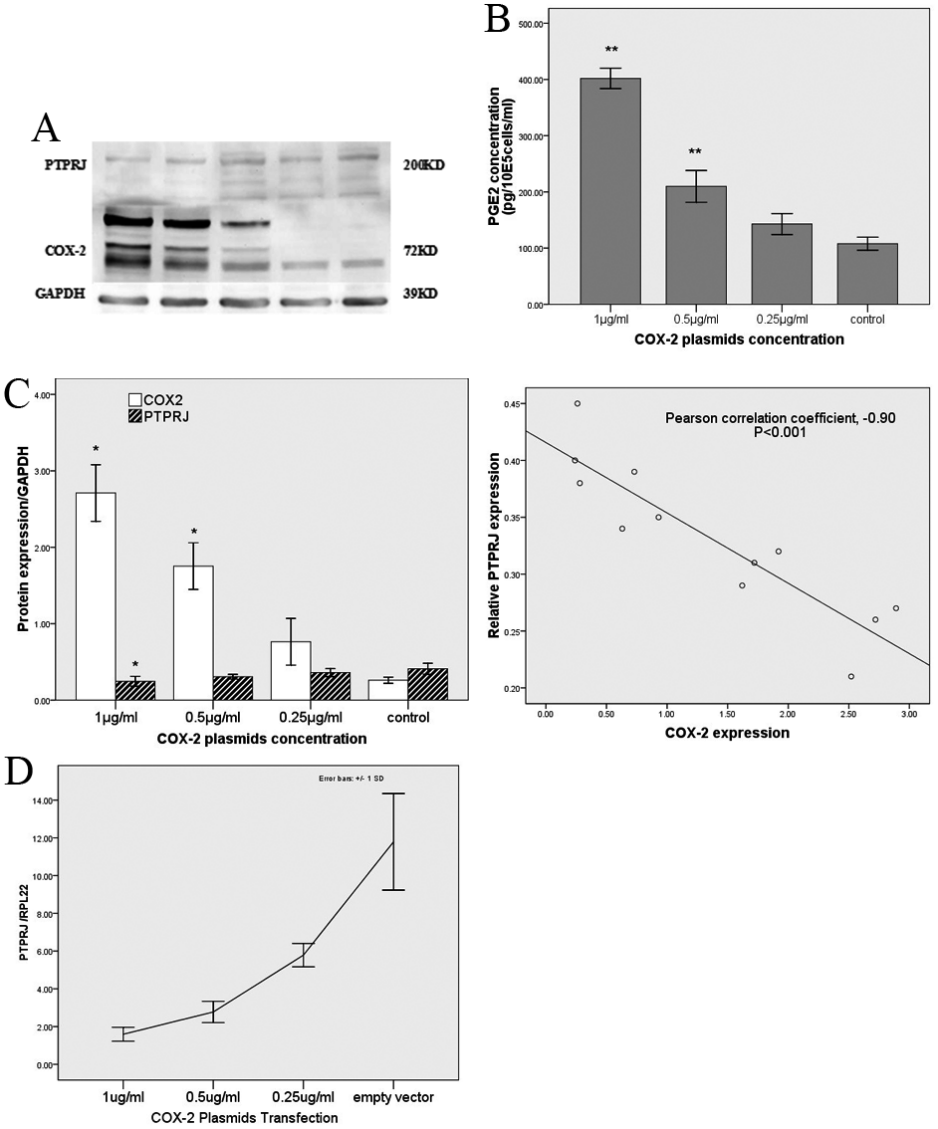
PTPRJ expression is down-regulated by COX-2 over-expression. (A) Western blot analysis of PTPRJ and COX-2 in protein extracts from HUVEC cells transiently transfected for 48 h with COX-2 plasmids. Expression is normalized to GAPDH. Relative protein levels were quantified using Image J. (B) ELISA quantification of PGE_2_ in the supernatant of HUVECs after transfection with COX-2 plasmids. The data is expressed as PGE_2_ concentration. ** P<0.01 versus control group. (C) Correlation of COX-2 and PTPRJ expression in HUVECs after transfection with COX-2 plasmids. (Pearson correlation coefficient, −0.90, P<0.01). (D) Relative PTPRJ expression in HUVEC cells transfected with varying concentrations of COX-2 plasmids and quantified by qRT-PCR. Values were normalized to PRL22 expression. *P<0.05 versus control group.

In an attempt to identify the role of COX-2/PGE_2_ signaling in the expression of PTPRJ, HUVECs were transfected with COX-2 plasmids at concentrations of 1 µg/ml, 0.5 µg/ml, or 0.25 µg/ml. At 48 hours post-transfection, COX-2 expression had increased by 10.44- (±0.70), 6.79- (±1.05), and 2.91- (±0.36) fold, respectively, compared with empty vector control (**Fig. 4A** ). The same COX-2 transfections also led to fold-increases of PGE_2_ levels in the culture media (3.77±0.57, 1.94±0.17, and 1.33±0.16, respectively) (**Fig. 4 B**). However, expression of PTPRJ decreased (0.60±0.08, 0.75±0.09, 0.88±0.04) (**Fig. 4** **C**) relative to the control. RT-PCR revealed that PTPRJ mRNA also decreased (0.15±0.03, 0.26±0.05, 0.47±0.09) (**Fig. 4** **D**). We found a significant correlation between COX-2 and PTPRJ expression (Pearson correlation coefficient, −0.90; n = 12; P<0.001, two-tailed; **Fig. 4** **E**).

We also transfected PTPRJ plasmids into HUVECs, however, no changes in COX-2 protein or mRNA expression were observed (*data not shown*).

### The COX-2 selective inhibitor Celecoxib rescues PTPRJ expression in HUVECs

**Figure 5:**
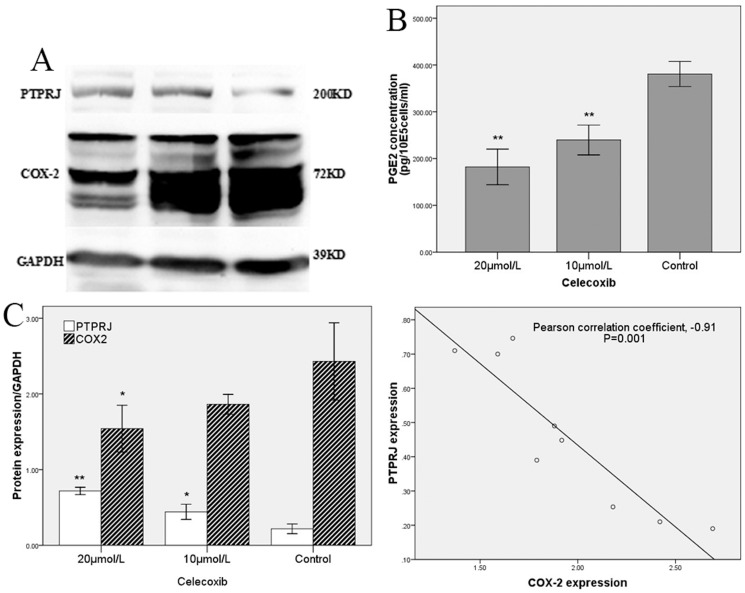
PTPRJ expression is rescsued by the COX-2 selective inhibitor Celecoxib. (A) Western blot analysis of PTPRJ and COX-2 in protein extracts from HUVEC cells stably over-expressing COX-2 and treated for 24 h with 20 µM or 10 µM Celecoxib. Expression is normalized to GAPDH. Relative levels of PTPRJ were quantified using Image J. (B) ELISA quantification of PGE_2_ in HUVEC supernatant; data is expressed as concentration of PGE_2_. ** P<0.01 compared to the 0.1% DMSO-treated control group. (C) Relative COX-2 and PTPRJ expression in HUVEC cells treated with varying concentrations of Celecoxib. Expression is normalized to GAPDH. Relative levels of COX-2 and PTPRJ were quantified using Image J. *P<0.05.

HUVEC clones with stable COX-2 over-expression were treated by the COX-2 selective inhibitor celecoxib at concentrations of 20 µM and 10 µM. At 12 hours after administration, the PGE_2_ level in the supernatant media had decreased significantly by 0.48-fold (±0.03) for the 20 µM treatment and 0.63-fold (±0.03) for the 10 µM treatment, compared to the 0.1% DMSO-treated control (Fig. 5 B). However, at the same time point, expression of PTPRJ had increased by 3.34- (±0.37) and 2.05- (±0.28) fold compared with control, respectively (Fig. 5A). A significant correlation between COX-2 and PTPRJ expression was also identified (Pearson correlation coefficient, −0.91; n = 9; P = 0.001, two-tailed; Fig. 5 D), suggesting that the expression of PTPRJ, which is down-regulated by COX-2 over-expression, was rescued by Celecoxib treatment.

## Discussion

PTPRJ is recognized as a tumor suppressor gene in colorectal carcinoma, breast tumors and even normal mammary epithelial cells [7], [29], but the expression and function of PTPRJ in endothelial cells during angiogenesis has remained unclear.

Our results demonstrated that the expression of PTPRJ both in vivo and in vitro is decreased during injury-induced neointimal hyperplasia. This is consistent with several previous studies [29], [30], which demonstrated that the expression of PTPRJ was generally decreased in proliferating and migrating cells during vessel repair compared with that in adjacent, quiescent endothelial cells.

Using bioinformatics, we found a negative correlation between PTPRJ and COX-2 expression in GSE39264, an array covering the whole genome transcriptional profile of purified mouse aortic endothelial cells. This result suggests that the expression of PTPRJ and COX-2 may be coordinated in endothelial cells.

Several significant studies have found that PTPRJ expression could be regulated by the pVHL-HIF2α axis, microRNA-328 and a long non-coding RNA species called Ptprj-as1 [30], [31]. In the context of atherosclerosis, vascular injury or inflammation, and tumor angiogenesis, COX-2/PGE_2_ signaling in endothelial cells is stimulated, which could indicate a major role in regulating PTPRJ expression and enzymatic activity [19], [32]. Consistent with these reports, using a rabbit carotid artery BI model in vivo, our results have elucidated that both expression of COX-2 and PGE2 is associated with PTPRJ expression. Using transfection of plasmids into HUVECs in vitro, our results also identified negative regulation of PTPRJ expression by both COX-2 and PGE2, which again emphasized the correlation of COX-2/PGE2 and PTPRJ signaling in endothelial cells. However, the precise relationship between the expression of COX-2 and PGE2 remains unknown.

Vascular endothelial cells have an important role on neointimal hyperplasia after balloon injury. Increasing evidence indicate that the endothelial cells which protect blood vessels can effectively inhibit smooth muscle cell proliferation and intimal hyperplasia [33], [34]. It has been shown that PTPRJ is mainly expressed vascular endothelial cells in the vessel, and has no expression in smooth muscle cells [35]. After balloon injury, as acute phase protein, COX-2 expression in vascular endothelial cells increased, while PTPRJ expression decreased, and COX-2 expression was negatively correlated with PTPRJ expression, which is the process of stress response in vascular endothelial cells after balloon injury, and is in line with the finding that COX-2 over-expression in vascular endothelial cells inhibited PTPRJ expression in in vitro experiments, while in in vitro experiments, inhibition of COX-2 function with celecoxib can restore PTPRJ expression, providing new ideas for the protection of endothelial cell function after balloon injury. Thus our in vitro and in vivo experiments corroborate each other.

Endothelial COX-2 can synthesize prostanoids both intracellularly and via transcellullar metabolism through crosstalk with neighboring cells, and prostanoids can also promote angiogenesis by facilitating migration and proliferation of ECs via activation of the PGE2 receptor and induction of VEGF [32], [36]–[39]. Here, our study revealed that in addition to the capacity to induce VEGF, COX-2/PGE2 signaling in ECs could promote EC motility by regulating PTPRJ expression. Together, these results demonstrated that interaction between COX-2/PGE2 and PTPRJ plays a critical role in pathological process involved in angiogenesis. The reason we focused on PGE2 is because it has previously been suggested to be involved in angiogenesis [40], [41], while the correlation of other PGs like PGI1 and TXA2 with angiogenesis was not found in these studies. Future studies are imperative on the regulation of downstream COX-2 mediators or related receptor expression on the expression of PTPRJ.

In summary, this work demonstrated that COX-2/PGE_2_ signaling was a negative regulator of PTPRJ expression in endothelial cells, thus revealing that through modulation of COX-2/PGE_2_ signaling, the expression and function of PTPRJ could be modulated both in vivo and in vitro. This, in turn, could affect endothelial cell migration and vessel remodeling. Further studies are needed to investigate the detailed pathway linking COX-2/PGE_2_ signaling to the expression of PTPRJ, which could be used as a therapeutic strategy in pathological angiogenesis.
